# Primary perianal alveolar rhabdomyosarcoma with uncommon metastatic sites: a case report and follow-up using ^18^F-FDG PET/CT

**DOI:** 10.3389/fmed.2024.1474698

**Published:** 2024-12-17

**Authors:** Yihan Yang, Zhao Chen, Yongkang Qiu, Jia Cheng, Ritai Na, Min Liu, Lei Kang

**Affiliations:** ^1^Department of Nuclear Medicine, Peking University First Hospital, Beijing, China; ^2^Department of Respiratory Medicine, University of Hong Kong Shenzhen Hospital, Shenzhen, China

**Keywords:** alveolar rhabdomyosarcoma, perianal, metastasis, ^18^F-FDG, PET/CT, case report

## Abstract

**Background:**

Rhabdomyosarcoma (RMS), a rare pediatric soft tissue neoplasm, predominantly develops in late childhood and adolescence with no discernible gender bias. Alveolar rhabdomyosarcoma (ARMS) stems from mesenchymal cells and may develop most frequently in the trunk, extremities, and head/neck areas, while occurrences in the pelvic cavity are less frequent. The manifestation is typically characterized by a high rate of aggressive metastasis and a poor overall survival prognosis.

**Case report:**

We present the case of an 11-year-old girl with ARMS initially presenting with a perianal mass. The diagnostic workup included MRI and PET/CT, which highlighted the tumor’s aggressive nature and metastatic potential. Subsequent metastases to the pleura, lymph nodes, and, less frequently, to the bone were detected using 2-deoxy-2-[fluorine-18]-fluoro-D-glucose positron emission tomography combined with computed tomography (^18^F-FDG PET/CT) imaging. The patient underwent a multi-regimen chemotherapy protocol but showed an incomplete response, indicating a poor prognosis.

**Conclusion:**

This report presents a rare case of ARMS primarily located in the perianal region with multiple metastases, including the uncommon occurrence of bone metastasis, which illustrates the challenges in diagnosing and treating ARMS, emphasizing the need for accurate and early diagnosis, advanced imaging for disease assessment, and more effective treatment strategies. ^18^F-FDG PET/CT imaging highlights its preeminence in detecting multiple rare metastatic lesions. However, the persistent disease activity despite treatment highlights the need for further research into the biology of ARMS and the development of novel therapeutics to improve patient outcomes.

## Introduction

RMS is an uncommon soft tissue malignancy that primarily affects pediatric patients (1). ARMS, the second most prevalent subtype of RMS, is distinguished by a unique arrangement of round-to-oval-shaped cells forming an alveolar pattern lined by fibrous septa and containing multinucleated, wreath-like giant cells. ARMS typically manifests during late childhood and adolescence, with no observed gender bias (2–4). ARMS stems from mesenchymal cells and may develop most frequently in the trunk, extremities, and head/neck areas, while incidence in the pelvic cavity is low (4). ARMS behaves aggressively in clinical terms, with 25–30% of patients exhibiting either distant or local metastasis through lymphatic or hematogenous routes (1, 5). The clinical manifestations of ARMS can vary depending on the location of the tumor, but common signs and symptoms include swelling, pain, and functional impairment. Pathological findings serve as the gold standard in the diagnosis of ARMS, with fluorescence *in situ* hybridization (FISH) being particularly significant.

In this report, we present a case of a girl diagnosed with ARMS primarily in the perianal region, who initially presented with a right-sided gluteal lump. With the development of various multimodal medical image fusion techniques (6–9), the accuracy of ARMS examinations is expected to improve over time. Currently, imaging modalities such as magnetic resonance imaging (MRI), computed tomography (CT), and ^18^F-FDG PET/CT, are commonly used. MRI is the optimal choice for detailed imaging when it comes to clarifying the location of lesions, especially soft tissue injuries, particularly ARMS in this case. CT scans have an advantage over MRI in detecting alterations in the bone structure (10). Surging ^18^F-FDG uptake revealed subsequent pleural, lymph node, and bone metastases. The incidence of bone metastasis is remarkably rare in ARMS. PET/CT has demonstrated its superiority in the setting of multiple metastatic tumors by demonstrating several lesions within a relatively short period and providing quantitative information without missing significant bone metastasis, especially in this case. Metabolic changes between bone metastases before and after chemotherapy were only detectable through ^18^F-FDG PET/CT.

### Case presentation

An 11-year-old girl presented with a mass on the right side of her buttocks for over a month with a slight weight loss of approximately 500 g recently. Physical examination revealed multiple palpable lymph nodes in the neck, left supraclavicular fossa, and groin areas, which were firm and mobile. The nodes in the groin were notably enlarged, measuring a maximum of approximately 2 cm × 2 cm. Perianal inflammation with a ruptured swelling on the right side of the buttocks covered by a white membrane was observed. Laboratory tests revealed elevated markers: CA199 (43.5 U/mL), CA125 (136.0 U/mL), CA72-4 (11.4 U/mL), and NSE (34.6 ng/mL).

**Figure 1:**
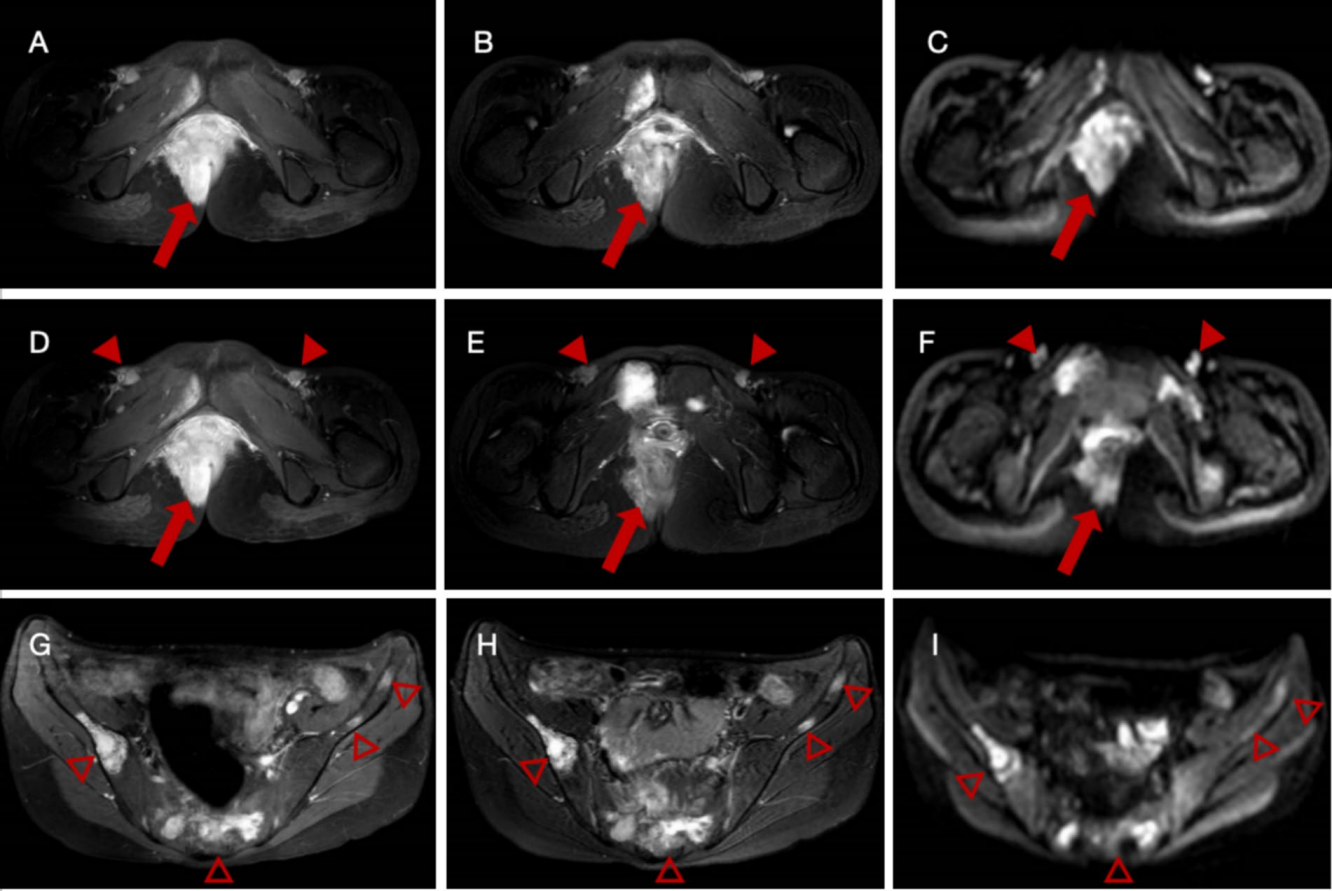
Enhanced pelvic magnetic resonance imaging (MRI) of a female patient with alveolar rhabdomyosarcoma with multiple metastases. **(A–C)** Images reveal a thickening of the soft tissue on the right side of the perineum and around the anus with significant enhancement (arrows), extending locally to the anal fissure. **(D–F)** Multiple enlarged lymph nodes (arrowheads) are observed in the bilateral inguinal regions. A noticeable increase in signal intensity with the short diameter of the largest node measured approximately 1.4 cm after enhancement. **(G–I)** Multiple nodular and patchy areas of low signal intensity on T1-weighted images (T1WI) and high signal intensity on fat-suppressed T2-weighted images (fsT2WI) are seen in the sacrum (hallow arrowheads).

Enhanced pelvic MRI demonstrated soft tissue thickening around the anus with a malignant-appearing mass invading the rectum, vagina, and right levator ani muscle, possibly originating from mesenchymal tissue (Figures 1A–F). Multiple abnormal signals were detected in the pelvic bones, suggesting a high possibility of metastasis. Nodular and patchy areas of low signal intensity on T1-weighted images (T1WI) and high signal intensity on fat-suppressed T2-weighted images (fsT2WI) were seen in the bilateral iliac bones, pubic bones, lumbar-sacral vertebrae, part of the accessory area, and the right femur. Diffusion-weighted imaging (DWI) showed high signal intensity (Figures 1G–I). After enhancement, a significant increase in signal intensity is observed, with soft tissue formation visible in the local upper limb of the pubis. Multiple enlarged lymph nodes were observed in the bilateral inguinal regions, also suggesting possible metastasis.

**Figure 2:**
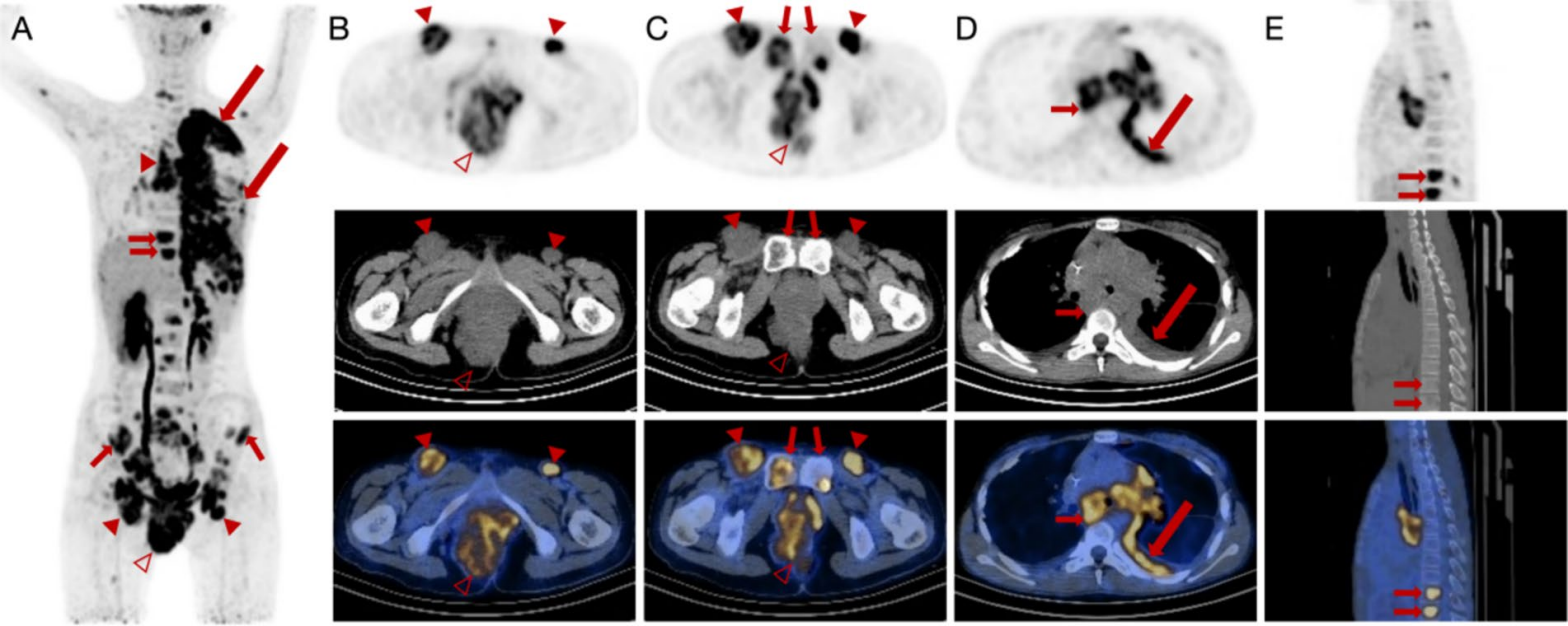
^18^F-FDG PET/CT images of a female patient diagnosed with alveolar rhabdomyosarcoma along with multiple metastases. **(A–C)** Anteroposterior three-dimensional maximum intensity projection (MIP) image demonstrates several increased metabolic activity sites. Enlarged lymph nodes (arrowheads) are present in the bilateral inguinal region, and the largest node has a short diameter of approximately 39 mm with increased FDG uptake (SUV_max_ = 8.9). An irregular soft tissue density mass (hallow arrowheads) is visible around the anus and on the right side of the perineum, measuring approximately 88 mm × 68 mm × 111 mm (AP × RL × SI) with increased FDG uptake (SUV_max_ = 48.7). **(D,E)** There is fluid accumulation in the left thoracic cavity and irregular thickening of the left pleura (long arrows) with increased FDG uptake (SUV_max_ = 8.3). Increased FDG uptake (SUV_max_ = 8.2) is manifested in the vertebrae, indicating bone erosions (short arrows).

To further locate lesion areas, an ^18^F-FDG PET/CT scan was performed. There was a mass in the perianal region with a maximum standardized uptake value (SUV_max_) of 48.7. In addition, multiple enlarged lymph nodes with increased glucose metabolism were present in the left neck, above the clavicle (SUV_max_ = 7.7), in the axilla (SUV_max_ = 3.7), mediastinum, both lung hilar regions (SUV_max_ = 7.9), bilateral inguinal regions, pelvis, and near the abdominal aorta (SUV_max_ = 8.9) (Figures 2A–C). A mass in the left lung (SUV_max_ = 6.5) was accompanied by thickening in multiple areas of the left pleura (SUV_max_ = 8.3). Increased bone glucose metabolism was observed throughout the body, with several areas of bone destruction noted (SUV_max_ = 8.2) (Figures 2D–E).

Biopsies of the perineal lesions showed infiltrations of medium to large atypical cells with alveolar, trabecular, and nest-like patterns within the interstitial cell, positive immunohistochemistry for vimentin, desmin, MyoD1, myogenin, and myoglobin, and a Ki67 index of 70%. Mitotic figures were readily observed with compressed and deformed tissue. FISH detected a 50% abnormal breakage of the FOXO1 (FKHR) gene. Pleural effusion cytology revealed numerous clusters of atypical cells with large and deeply stained nuclei and a high nuclear-to-cytoplasmic ratio. Immunohistochemical staining showed positive reactions for desmin, MyoD1, myogenin, and myoglobin. The pathological results were consistent with RMS. The patient was subjected to a multi-regimen chemotherapy protocol, which included VAC (vincristine, cyclophosphamide, and dactinomycin), VDC (vincristine, cyclophosphamide, and doxorubicin), and IE (ifosfamide and etoposide), but showed incomplete eradication after 4 months of treatment, indicating a poor prognosis.

**Figure 3:**
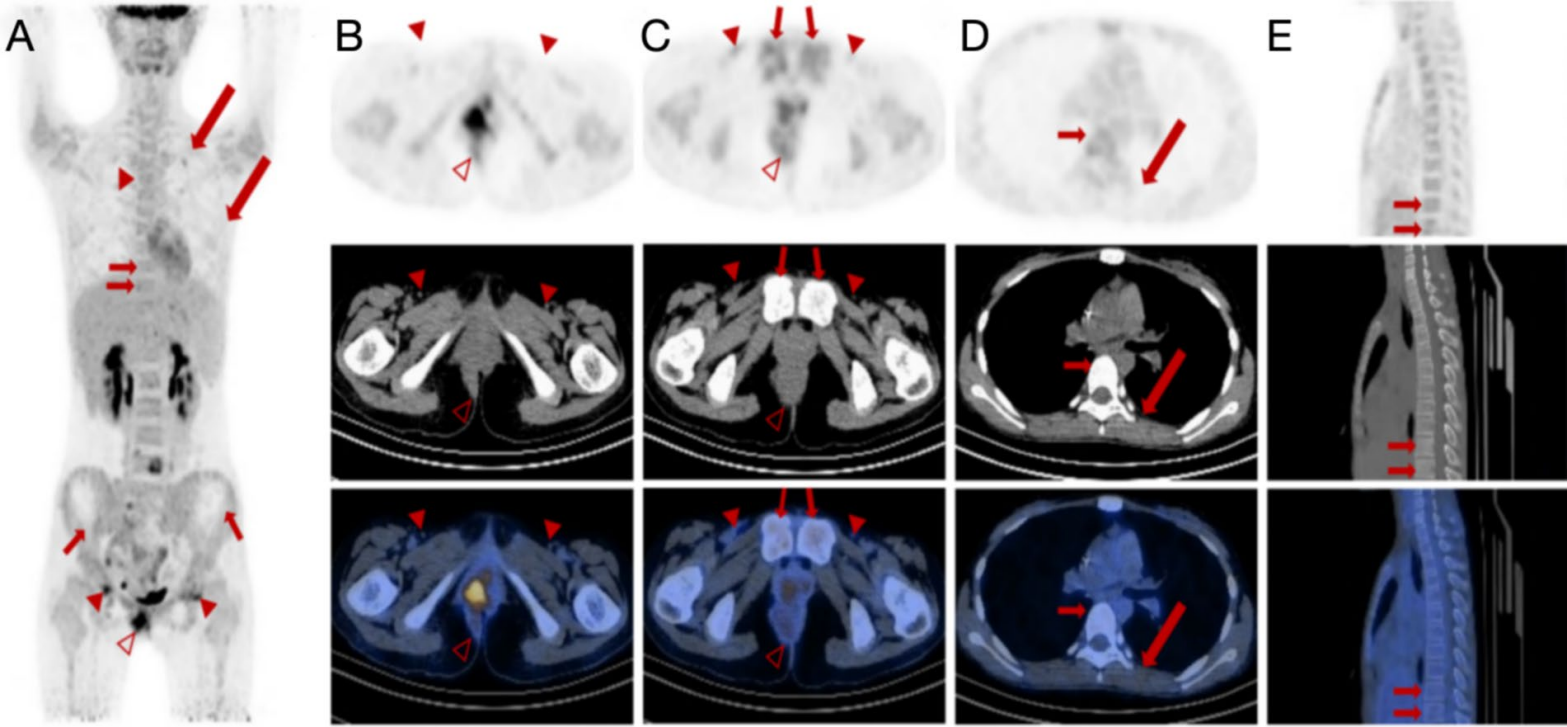
^18^F-FDG PET/CT images of a female patient diagnosed with alveolar rhabdomyosarcoma along with multiple metastases after chemotherapy. **(A–C)** Anteroposterior three-dimensional maximum intensity projection (MIP) image demonstrates several increased metabolic activity sites. Multiple lymph nodes (arrowheads) remain visible, with the largest lymph node manifesting a short diameter of approximately 39 mm to 10 mm. The lymph nodes next to the right iliac vessel and in the bilateral inguinal areas show increased FDG uptake with SUV_max_ of 4.2. The irregular soft tissue density mass (hallow arrowheads) around the anus can be seen in the perianal and perineal areas, measuring approximately 55 mm × 52 mm (AP × RL) with a remarkable SUV_max_ decrease of 9.5 compared to 48.7 before chemotherapy. **(D,E)** The original bilateral pleural effusion (long arrows) disappeared. Multiple focal FDG uptake lesions can be seen in the L4 and L5 vertebral plates with SUV_max_ ranging from 8.2 to 5.5 (short arrows). However, multiple skeletal lesions with diffusely increased glucose metabolism are still observed throughout the rest of the body.

This patient demonstrated a degree of control over the tumor in terms of the efficacy of chemotherapy. However, it did not result in the complete eradication. The mass demonstrated a reduction in size compared to previous measurements, accompanied by a decrease in metabolic activity. The volume of the enlarged lymph nodes decreased with reduced metabolic activity (Figures 3A–C). The lesions in the L4 vertebral plate, L5, sacrum, right pubic bone, and bilateral acetabulum showed a decrease in metabolic activity compared to previous measurements and an increase in bone density (Figures 3D,E). Notably, the lesion with increased glucose metabolism at L5 was a new development. Other lesions demonstrated a return to normal metabolic levels. Conversely, multiple skeletal lesions with diffusely increased glucose metabolism were observed throughout the rest of the body (Figure 3A).

Despite partial tumor control, the patient’s lesions did not fully resolve. The mass and lymph nodes showed reduced size and metabolic activity, with some bone lesions showing increased bone density. However, new and persistent skeletal lesions with increased glucose metabolism were observed, indicating ongoing disease activity.

## Discussion

ARMS, the most undifferentiated subtype of RMS, is characterized by a uniform population of primitive round cells that typically lack visible signs of skeletal muscle differentiation. It is known for its aggressiveness and is found predominantly in the trunk and extremities (4). Conversely, ARMS in the pelvic cavity is rare, with symptoms mainly including perianal masses and pain resembling inflammation (10, 11), frequently leading to a misdiagnosis of perianal abscess. Thus, confirming a diagnosis of ARMS generally necessitates pathological examination exhibiting a central reduction in cell cohesion and the development of irregular alveolar spaces.

The occurrence of bone metastases in ARMS is exceedingly uncommon. However, unusually extensive bone erosions were present in this case. While CT provides high-resolution imaging for ARMS cases, its use of ionizing radiation raises concerns about inducing secondary cancers, especially when examining children who have already demonstrated a predisposition to developing malignancies at an early age (12, 13).

While RMS appears largely non-specific on MRI, it is the ideal choice for preliminary evaluation of local tumor assessment and its relationship to adjacent organs and peripheral neurovascular structures. The lesion typically presents with intermediate signal intensity on T1-weighted images and intermediate to high signal intensity on T2-weighted images such as the majority of soft tissue tumors (11). Nonetheless, MRI can be overly sensitive for cancer diagnosis, as it can produce imaging responses to inflammation or small masses.

The staging of ARMS is crucial for guiding the selection of subsequent treatment strategies. PET/CT excels in identifying multiple metastatic lesions and providing quantitative information, making it particularly effective and accurate in identifying lymph node involvement and distant metastases (14, 15). During a PET/CT procedure, the CT scan is specifically designed to identify anatomical structures, offering more detailed information. However, the high radiation dose of PET/CT (15) raises concerns about the potential of provoking secondary cancer. In previous case reports, ^18^F-FDG PET/CT imaging in ARMS patients has been mostly performed in nasal sinuses (16), retroperitoneum (17), and prostate (18), with rare mention of perianal occurrences. Perianal lesions in adolescent patients are often associated with inflammatory conditions such as Crohn’s disease and anal fistulas. The significance of ^18^F-FDG PET/CT lies in its ability to detect metastatic lesions and provide important insights for the qualitative diagnosis of tumors.

The metabolic activity in RMS, attributed to the Warburg effect, involves the upregulation of the GLUT4 gene and activation of the receptor tyrosine kinase (RTK) pathway, which, in turn, contributes to an escalation in glucose metabolism (17). It is noteworthy that an inflammatory response can also induce increased glucose uptake, potentially manifesting as lymphadenopathy. In two instances, the histological appearance mimicked that of a small round-cell tumor, particularly ARMS, under light microscopy. Muscle regeneration and proliferative myositis are reactive conditions secondary to insult and may occasionally coexist (19). This could influence the diagnostic process for ARMS from physical examination to pathological evaluation. Particularly in this case, an increased glucose uptake was also observed in the right thigh muscle. With the tumor demonstrating multiple metastases, the assessment of muscle regeneration, inflammatory response, and authentic tumor lesions could influence the selection of therapeutic strategies, subsequently affecting treatment progression and potentially exerting a detrimental effect on prognosis. Therefore, distinguishing between inflammation and neoplasia is of paramount importance. It is essential to comprehensively evaluate the metabolic information from PET/CT, the morphological characteristics from CT, the patient’s clinical history, and other ancillary test results to accurately differentiate between inflammation and neoplasia before making a diagnosis.

^18^F-FDG PET/CT serves as a referential modality in the preliminary differentiation of pathological entities. PET/CT operates by assessing the uptake of radiotracers across various anatomical regions. A heightened accumulation may suggest a higher probability of malignancy. In cases where the metabolic rates of inflammation and neoplasia are similar, morphological characteristics, among others, can help differentiate between the two. The SUV_max_ and other single-pixel SUV values are frequently utilized as a quantitative measure of tumor metabolic activity due to their straightforward integration into imaging software, facilitating ease of use. A study demonstrated that patients with SUV_max_ < 9 exhibited significantly improved 3-year progression-free survival rates (62% for SUV_max_ < 9 vs. 39% for SUV_max_ ≥ 9, *p* = 0.02) (17, 20). In our case, the patient’s SUV_max_ of 48.7, along with several other lesions showing SUV_max_ of approximately 9, indicated a more unfavorable prognosis. Nonetheless, this semi-quantitative assessment is prone to variability caused by numerous biological and technical influences, including patient weight, blood glucose levels, and PET scan parameters such as uptake duration and calibration accuracy (21, 22). Concurrently, a thorough integration of the patient’s medical history, corroborative imaging findings, and results from objective investigations is imperative for a holistic analysis. Metabolic tumor volume (MTV), which refers to the sum of the primary tumor and the largest distant lesion or the metabolic volume of the primary tumor, is considered a superior prognostic factor (23). In this case, the MTV specifically exceeded the threshold of 200 cm^3^, implying extremely intense malignancy.

Considering the potential hazards of ionizing radiation exposure in pediatric patients, there has been a paradigm shift from CT to MRI (23). The advantages of PET/MRI over PET/CT are substantial, encompassing decreased radiation exposure, reduced instances of sedation and general anesthesia, consolidated single-day visits, and the concurrent utilization of two standalone advanced imaging techniques that are pivotal for staging and evaluating treatment response in pediatric oncology. Furthermore, there is a prospective enhancement in parent and patient satisfaction due to the convenience of a single visit (24). Regrettably, due to constraints in our diagnostic equipment, we were unable to conduct a PET/MRI scan. It is hypothesized that PET/MRI imaging would have offered superior delineation in this case with extensive soft tissue involvement. The radiation exposure inherent to PET/CT is unavoidable. However, we can extrapolate the outcomes of a combined PET/MRI procedure based on the structural data provided by the CT scan. This approach offers a practical solution to the limitations imposed by equipment constraints and serves as a supplement to the scarcity caused by the high cost of equipment.

In clinical practice, it is of paramount significance to meticulously evaluate the extent of surgical intervention. More extensive surgical excisions often necessitate the sacrifice of normal function and can lead to postoperative complications that significantly impact the patient’s quality of life. Conversely, overly conservative surgery may result in local recurrence and a decrease in overall survival. Radioactive therapy encompasses both brachytherapy and radiation therapy. It is primarily used as adjuvant therapy or neoadjuvant therapy if the tumor has not metastasized to other organs (10). Given the extensive impact of the tumor in this case, both surgery and radiation therapy have been ruled out, thus opting for chemotherapy. The patient exhibited a measurable response to chemotherapy, indicating partial control of the tumor. However, complete eradication was not achieved.

ARMS is classified as an intermediate-to high-risk subtype of RMS according to the risk stratification system of the Children’s Oncology Group-Soft Tissue Sarcoma (COG-STS) and carries a less favorable prognosis. The estimated 5-year survival rate for intermediate-risk ARMS ranges from 65 to 73%, while high-risk lesions have a significantly lower 5-year survival rate of less than 30% (5). Although the 5-year survival rate for patients with low-risk disease has nearly reached 90%, the overall survival rate for children with metastatic disease remains concerningly low, at 25–30% at 3 years (10, 25–28). This is the case even with the administration of high-dose chemotherapy and stem cell rescue treatments. Based on the statistical data and the patient’s condition in this case, it is inferred that the patient’s prognosis is likely to be poor. Even after undergoing aggressive chemotherapy, the impact of altering the prognosis is minimal, validating the previous hypothesis.

Despite the rapid advancements in medicine, there remains a significant deficiency in the exploration of therapeutic strategies for alveolar rhabdomyosarcoma (ARMS). A recent real-world study has revealed that metronomic maintenance therapy (MMT) has significantly improved the survival of patients with high-risk RMS in clinical trials. However, there remains a lack of relevant data on its effectiveness in real-world situations. There is an urgent need for further clinical practice and therapeutic modalities (29).

## Conclusion

ARMS remains a formidable challenge in pediatric oncology due to its aggressive nature and propensity for metastasis. Given the clinical and pathological manifestations of ARMS, it can be misinterpreted as an inflammatory lesion or a result of muscle trauma. PET/CT has been proven to be highly effective in the precise staging and prognostic evaluation of ARMS, and is particularly adept at identifying lymph node involvement and detecting the dispersion of distant metastases, whose capabilities are crucial in the management of ARMS. Therefore, PET/CT not only aids in the accurate diagnosis of ARMS but also contributes significantly to the development of an effective treatment strategy and prognosis. Considering the superior soft tissue contrast provided by MRI, PET/MRI may offer enhanced diagnostic accuracy for ARMS compared to PET/CT. This case underscores the critical need for precise and timely diagnostic procedures, the invaluable role of ^18^F-FDG PET/CT in comprehensive disease evaluation, and the imperative for the development of more potent therapeutic strategies to combat ARMS effectively. Further research into the biology of ARMS and the development of novel therapeutic approaches is warranted to improve the outcomes for patients with this devastating disease.

## Data Availability

The original contributions presented in the study are included in the article/supplementary material, further inquiries can be directed to the corresponding author.
